# Multitasking while studying – grit moderates the relationship of situational motivation and multitasking

**DOI:** 10.3389/fpsyg.2024.1404767

**Published:** 2024-07-16

**Authors:** Olga Bachmann, Carola Grunschel, Stefan Fries

**Affiliations:** ^1^Department of Psychology, Bielefeld University, Bielefeld, Germany; ^2^Department of Psychology, University of Münster, Münster, North Rhine-Westphalia, Germany

**Keywords:** students, multitasking, motivation, self-determination, grit

## Abstract

Multitasking during studying is frequent among students. In this experience sampling study we examine if multitasking during studying can be explained by situational study motivation and the personality trait grit; and if grit moderates the relationship of situational motivation and multitasking. Eighty-eight students participated. All participants planned to write an important exam within the upcoming 2 weeks. Situational motivation was conceptualized along the lines of self-determination theory, differentiating between autonomous and controlled motivation. Also, we assessed students’ grit. Hypotheses were tested using multilevel modeling in MPlus. As predicted, students multitasked less when situational study motivation was autonomous (vs. controlled). Contrary to predictions, we did not find a significant main effect of grit on multitasking. However, the interaction effect was significant, indicating that in situations with relatively controlled study motivation grittier students are more likely to refrain from multitasking than their less gritty peers.

## Introduction

Evidence, from both research and everyday observations, suggests that multitasking is prevalent among students. Research shows that students engage in additional activities such as checking their social media or talking to their peers more than a third of their study time (Kraushaar and Novak, 2010; Bachmann et al., 2018). Multitasking can be concurrent, like listening to a lecture and simultaneously talking to a peer, or sequential, where a student pauses their main task (reading a text book) to complete an additional activity (checking social media) before returning to the main task (e.g., Salvucci et al., 2009; Reinecke et al., 2018). Within multitasking, particularly when activities are performed concurrently, a student’s focus is necessarily divided between the tasks. Therefore, multitasking brings along negative consequences for learning and performance such as declines in note-taking, test performance and even GPA (Bellur et al., 2015; May and Elder, 2018). So the question arises: Why do students multitask and what can help balance this pervasive behavior?

### Does motivation predict multitasking?

While multitasking is influenced by a wide variety of factors, one potential reason why students multitask during studying relates to the motivation that drives studying (Wang and Tchernev, 2012; Calderwood et al., 2014). Several studies have shown that autonomous motivation for studying is linked to more successful self-regulation in academia compared to controlled motivation, with autonomous motivation predicting less procrastination, more persistence, stronger engagement and better concentration (Vallerand et al., 1993; Vansteenkiste et al., 2004; Cavusoglu and Karatas, 2015; Froiland and Worrell, 2016). In fact, this pattern of results has been demonstrated so widely, that controlled motivation is frequently used synonymously with suboptimal motivation and autonomous motivation synonymously with optimal motivation (Ryan and Deci, 2017; Grund and Fries, 2018; Slemp et al., 2020). Why autonomous motivation is linked with so many academic advantages, can be explained within the framework of self-determination theory (SDT) (Ryan and Deci, 2017, 2020). SDT states that all people have an innate desire to grow and develop, to connect with others, to understand and to learn. However, for this desire to unfold in real life, people need a context in which their basic psychological needs of relatedness, competence and autonomy are met. SDT focuses particularly on the need of autonomy: When the need for autonomy is satisfied, people are more likely to feel motivated from within themselves, i.e., autonomously. Autonomous motivation or autonomous regulation describes a state, in which people engage in an activity because they enjoy doing it (i.e., intrinsic motivation) or they find it important (i.e., identified motivation), whereas controlled motivation describes regulatory forms related to internal or external pressures (i.e., introjected or external motivation). People tend to have several or even all of these different motivational forms for their activities, but in different intensities. Therefore, most studies assess all of the before mentioned motivational forms to calculate one relative autonomy index, thereby conceptualizing autonomous and controlled motivation as two ends of the same continuum (Ryan and Deci, 2017).

As mentioned before, motivation on the autonomous end of this continuum has been linked to several favorably outcomes within the academic sphere (e.g., Vansteenkiste et al., 2017). Yet, the relationship of study motivation and multitasking has not been investigated empirically from a SDT perspective, although many authors speculate that multitasking may be driven by suboptimal motivation (e.g., Judd and Kennedy, 2011; Adler and Benbunan-Fich, 2013; Judd, 2013). We want to start closing this research gap by investigating if situational study motivation links to multitasking. We focus on study motivation on the situational level, because earlier research shows that (study) motivation fluctuates, depending on several factors such as learning content or alternative activities (Dietrich et al., 2017; Capelle et al., 2022). Furthermore, multitasking, e.g., quickly checking the phone while studying, is a relatively transitory behavior and people have difficulties remembering these short-lived multitasking episodes in retrospect (Kraushaar and Novak, 2010; David et al., 2015). To investigate study motivation as well as multitasking in a specific moment in time, we use experience sampling; thereby following recommendation from Wigfield and Koenka (2020) to use more situational approaches in motivation research (Ebner-Priemer and Trull, 2009; Bachmann et al., 2018).

In addition to investigating the connection of situational motivation and multitasking, it remains interesting to identify trait factors that could also relate to multitasking. This is particularly true as studying often includes pursuing goals that are externally provided and are therefore not well suited to stimulate autonomous motivation. We promote the view that teachers should support autonomous motivation wherever possible, in order to improve affective experience, focus, and performance (e.g., Katz et al., 2014; Ryan and Deci, 2017; Reeve and Cheon, 2021). However, we also accept that in reality students often study because of controlled reasons (Bachmann et al., 2024). Therefore, it is relevant to understand which personality traits are suitable to help students keep their focus on studying, even when situational motivation is controlled.

### Does grit predict multitasking?

One personality trait that seems like a promising candidate to relate to multitasking during studying is grit. Grit can be defined as a trait that captures “passion and perseverance for long-term goals” (Duckworth and Seligman, 2017, p. 716) and was conceptualized in order to grasp more about the noncognitive aspects that are necessary for professional success (e.g., Seligman, 2012). Grit is strongly related to self-control, statistically as well as conceptually. People who score high on trait self-control also tend to score high on grit with *r*s above 0.6 (Duckworth et al., 2007). However, whereas self-control describes the capacity to shield all kinds of goals from temptations, grit aims at acting toward a superordinate goal despite inner or outer hindrances, for example completing law school (Duckworth and Gross, 2014).

Pursuing a long term goal always includes accomplishing many smaller steps on the way. For example, completing a degree requires passing exams, which then necessitates studying, which again can be broken down in many smaller segments such as opening a book etc. (Kruglanski et al., 2002; Fujita et al., 2006; see also literature on deliberate practice, e.g., Ericsson et al., 1993; Duckworth et al., 2011). As gritty people are especially good in long-term goal pursuit, they may also be good in completing necessary lower-order goals, such as focusing on study activities without getting preoccupied by other activities. Ralph et al. (2017) support this reasoning. They investigated the relationship of grit and mind wandering during a university lecture and found that university students who scored higher on grit also reported less mind wandering. Although not the same, mind wandering is a concept somewhat related to multitasking in that both, multitasking and mind wandering are relatively short lived phenomena. Also, both describe that attentional resources are taken away from the main activity (e.g., studying) and are directed toward thoughts (mind wandering) or activities (multitasking) unrelated to the current activity, and both are related to declines in performance (Wammes et al., 2016).

### Does grit moderate the relationship of motivation and multitasking?

Grit is defined as perseverance and passion in the light of difficulties (Duckworth and Seligman, 2017) and, in the context of studying, one common difficulty is suboptimal motivation (Katz et al., 2014; Bachmann et al., 2018). Therefore, the question arises, if grit can cushion some of the impacts of suboptimal motivation. Moles et al. (2017) investigated in an experiment how grit interacted with ego-involving feedback promoting controlled motivation. The authors show that ego-involving feedback affected students low in grit particularly strongly, whereas grittier students were impacted less. These results indicate that grit may act as a protection against the negative impact of ego-involving feedback, thereby illustrating the interplay of situational motivation with grit. In the current study, we are interested if grit and situational motivation may also interact in predicting multitasking, thereby investigating a trait-situation interaction (e.g., Bonanno and Burton, 2013; Rottweiler et al., 2018; Miele et al., 2020).

### Research question

In the current study we aim to get a clearer understanding of why students multitask, thereby focusing on situational motivation and the personality trait grit. All participants were university students who were planning to write an exam within the next 2 weeks after the beginning of the ESM phase, which, so we assumed, would make the situations in which students studied relatively numerous. In our first hypothesis (H1) we predict that situational study motivation predicts multitasking likelihood. More precisely and coherent with self-determination theory (Ryan and Deci, 2017), we hypothesize that in situations where students are motivated autonomously for their current study activity, multitasking is less likely compared to situations in which students are motivated in a controlled way. Secondly (H2), we predict that higher levels of grit are associated with less multitasking during studying. We assume that grit is not only predictive for the successful pursuit of long term goals but also for focusing on moment-to-moment studying, which of course is a prerequisite for reaching the long term goal of completing a degree (for similar reasoning see Ralph et al., 2017). The final and central hypothesis (H3) relates to the interplay between situational motivation and grit in predicting multitasking. We predict an interaction between grit and motivation, with grit attenuating the relationship of motivation and multitasking. In other words, we predict that gritty students refrain more from multitasking in situations with motivational difficulties in form of controlled motivation compared to their less gritty peers.

## Materials and methods

### Ethics statement

This study conformed to all ethical standards of the German Psychological Association and the German Professional Association of Psychologists (Deutsche Gesellschaft für Psychologie und der Berufsverband Deutscher Psychologinnen und Psychologen e.V., 2004). The study was approved by the Ethics Committee and the Data Protection Office of Bielefeld University.

### Participants

A total of *N* = 88 students from a mid-sized German University participated in the study. Students were on average 21 years old (ranging between 18 and 33, *SD* = 2.5), 69 students (78.4%) were female. All students studied either law (*N* = 43) or education science (*N* = 45), were in the first semester and were planning to write an important exam within the next 2 weeks after the beginning of the ESM phase. All data were collected prior to the covid pandemic. All participants were recruited during a lecture or over the faculties’ mailing lists. Participants received 5 prompts per day over the course of 1 week. Overall, 3,080 prompts were signaled and 2,825 prompts were completed, leaving us with a compliance rate just of over 91%.

### Procedure

Students participated in an introductory session in small groups. An experimenter briefed participants on the experience sampling method (e.g., trying to answer as many prompts as possible, but not answering during driving). Then, participants received a smartphone device (Motorola Moto E) with the ESM software (movisensXS Version 0.8.4208) installed on it for the duration of the study. Participants chose if they wanted to receive prompts between 8 am and 8 pm or between 10 am and 10 pm, 58 participants chose the earlier option. Participants made themselves acquainted with the smartphone, filled in an example ESM prompt and were given the opportunity to ask questions. Then, participants filled in a questionnaire on the computer, which included grit, several other trait measures, and demographics. Participants were reminded on the starting point of the ESM phase, thanked and dismissed.

The ESM phase started less than 1 week after the introductory session, lasted for 7 days and was set in July, shortly before the exam period started. Participants received 5 prompts per day, whereby every prompt was signaled using an alarm that lasted up to 35 s and that was repeated up to five times in the consecutive 10 min after the first alarm. Whenever an ESM questionnaire was not completed after 15 min, this questionnaire was counted as missed. Participants were instructed to answer the ESM according to the situation in which the alarm was first signaled.

Between 1 and 2 weeks after the end of the ESM phase participants came back to the laboratory in small groups, filled in further questionnaires, handed back their study-phone and received their monetary compensation (50€ when participants had completed 80% or more of all signals, 30€ when less than 80% were completed).

### Measures and variables

This study was part of a larger data collection effort and included a variety of measures. In the following, only measures of relevance for the current endeavor are explicated, reporting them in the same order as they were presented in the study.

#### Grit

In the introductory session, all participants answered the Short Grit Scale (Grit-S) (Duckworth and Quinn, 2009). Grit-S was validated with a variety of populations and was frequently used since then (Pate et al., 2017; Alhadabi and Karpinski, 2019; Daniels et al., 2021). The scale consists of eight items; example items include “I finish whatever I begin,” and “New ideas and projects sometimes distract me from previous ones” (the latter being reverse coded). Participants answered all items on a Likert scale ranging from 1 (“not at all like me”) to 5 (“very much like me”). The scale was translated in German by the authors of the current study, translations can be received from the first author.

#### Activities

##### Main activity

In every ESM questionnaire participants were asked “What was the main activity you were doing right before the alarm?.” To answer this question, participants chose one out of six main options (“study,” “leisure,” “routines,” “part-time job,” “commute,” “other obligations”). After choosing one of the main categories, participants were asked to specify further what they did. For example, when the main activity study had been selected they could choose between categories such as “lecture,” “seminar/tutorial,” “study group,” preparation for an exam,”…. (For a similar procedure see Bachmann et al., 2018; Capelle et al., 2022). As we are interested study activities, only prompts with the answer “study” as the main activity were included in further analyses.

##### Additional activity

After the main activity was selected, participants were asked if there was an additional activity that they were engaged in right before the alarm. Participants could choose between the six activity options described above (study, leisure etc.) and the option “no additional activity.” If an additional activity was selected, participants were asked to further specify the activity (see above). Whenever participants selected an additional activity, this instance was coded as multitasking (multitasking = 1), when the option “no additional activity” was selected, this instance was coded as monotasking (multitasking = 0).

##### Filler items

If participants answered that they did not engage in any additional activity, they received other ESM questions which are irrelevant to this study. This was done in order to balance the length of ESM questionnaires, independent of the answers given.

#### Situational motivation

After indicating any additional activity in the ESM questionnaire, participants completed an autonomy measure. We asked participants why they engaged in their current main activity and gave them four potential reasons; “because I like doing it” related to intrinsic reasons, “because I find it important” to identified reasons, “because I should” to introjected, and “because I must” to external reasons (Reis et al., 2000; Grund et al., 2015). Participants rated each reason on a Likert scale ranging from 1 (does not fit at all) to 7 (fits exactly).

The four answers were merged into one autonomy score (AS; Reis et al., 2000; Grund et al., 2015). This was done using the equation: (intrinsic*2 + identified) − (introjected + external*2). The autonomy score ranges from −18 to +18, with negative numbers indicating controlled motivation and positive numbers indicating autonomous motivation.

### Data analysis

We used SPSS for all data processing, descriptive statistics and post-hoc analyses of the interaction effect (SPSS for Windows, Version 27.0). For multilevel analyses we used MPlus (Muthén, 2017).

Every participant answered several ESM measures over the course of 1 week. Consequently, the situations (Level 1) are nested within people (Level 2), thus observations are not independent of each other. Therefore, multilevel analyses were warranted in order to take into account that the presented data are hierarchical in nature. As the outcome variable multitasking is dichotomous, logistic multilevel regression analyses were conducted using the maximum likelihood estimator, which is standard in MPlus (Geiser, 2011; Muthén, 2017). Overall three models were tested, one model for each hypothesis.

## Results

### Descriptive statistics

In 930 situations out of the recorded 2,825 situations, “study” was reported as the main activity, indicating that students engaged in study activities at around 33% of all recorded situations. As the current manuscript focuses on situations in which students engage in study activities, further analyses include these 930 study situations. All 88 participants recorded at least one study activity. Of all reported study activities, students reported in 288 cases to engage in at least one other additional activity next to their main activity of studying (31% multitasking). Table 1 gives an overview on all additional activities. In most multitasking instances, students engaged in additional activities from the leisure domain that were social and/or media related. Therefore, the leisure domain is presented in greater detail.

**Table 1:** Frequencies of additional activities during studying.

Additional activity	*N*	%
Study	38	13
Leisure	187	65
Social Media/Text Message	75	40
Talking on the phone	3	2
Talking	35	19
Playing games on computer/phone	3	2
Reading	7	4
Watching something (e.g., TV)	12	6
Listening to music/radio	37	20
Going out	0	0
Doing sports or engaging in a hobby	0	0
Daydreaming	5	3
Napping	2	1
Sexual activity	0	0
Something else	8	4
Routines	47	16
Part-time job	5	2
Commute	5	2
Other obligations	6	2

On average participants reported an autonomy score slightly above the scale’s midpoint (*M* = 0.42, *SD* = 7.5), whereby the whole range was used (range: −18 – +18). In our sample, participants reported to be relatively gritty, with the average grit score being above the scale’s midpoint (*M* = 3.31, *SD* = 0.62), ranging from 1.25 to 4.75.

### Hypothesis testing

Results for all hypothesis tests are depicted in Table 2.

**Table 2:** Results from the logistic hierarchical regression analyses.

	Model 1: Multitasking on Autonomy Score			Model 2: Multitasking on Grit			Model 3: Multitasking on Autonomy Score and Grit, incl. Cross-level- interaction		
	*B*	*SE*	*p*	*B*	*SE*	*p*	*B*	*SE*	*p*
Fixed effects									
Threshold	0.894**	0.149	<0.001	0.880**	0.146	<0.001	0.920**	0.148	<0.001
Autonomy score	−0.038*	0.016	0.015				−0.029*	0.014	0.037
Grit				−0.33	0.267	0.213	−0.259	0.274	0.344
Autonomy score × Grit							0.048*	0.022	0.028
Variance components									
Autonomy score	19.381**	2.309	<0.001				56.094**	7.094	<0.001
Grit				0.379**	0.062	<0.001	0.379**	0.062	<0.001

Multitasking is Regressed on Autonomy Score (Model 1), Grit (Model 2) and on the Interaction of Autonomy Score and Grit (Model 3). **p < 0.001, *p < 0.05.

#### Model 1: situational motivation and multitasking

In H1 we predict that multitasking is less likely when the situational autonomy score is higher. In order to test this hypothesis we used the autonomy score as a Level 1 predictor, whereby the autonomy score was group mean centered (Bryk and Raudenbush, 1992; Geiser, 2011).

The significant and negative coefficient indicates that higher (positive) autonomy scores are, as predicted, associated with a decreased probability of multitasking (*B* = −0.038, *SE* = 0.016, *p* = 0.015). The random effect was significant, indicating that the association between the autonomy score and the slope differs between persons (*B* = 19.381, *SE* = 2.309, *p* < 0.001). We therefore retain H1.

#### Model 2: grit and multitasking

In H2 we predict that multitasking is less likely when the trait measure of grit is higher. In other words, we predicted that grittier students multitask less when studying. As grit is a predictor on the person level, grandmean centering was employed (Bryk and Raudenbush, 1992; Geiser, 2011).

The effect does not yield significant, the negative coefficient indicates that non-significant relationship points in the expected direction (*B* = −0.033, *SE* = 0.267, *p* = 0.213). Again, the variance component implies that this non-significant effect is different between people (*B* = 0.379, *SE* = 0.062, *p* < 0.001). Therefore, we reject H2.

#### Model 3: interaction of grit and motivation to predict multitasking

In our third hypothesis we predict a cross level interaction between the autonomy score and grit, with grittier students multitasking less in study situations with controlled motivation than their less gritty peers. This hypothesis is tested in Model 3. The autonomy score is a predictor on Level 1, grit is a predictor on Level 2 and an interaction term tests for a cross-level interaction between the autonomy score and grit; as before, multitasking acts as a dichotomous outcome variable. In order to maintain the variance on the person level and following recommendations, both predictor variables were grandmean centered (Geiser, 2011; Nezlek, 2012).

The results further confirm the negative relationship of autonomy score and multitasking (*B* = −0.029, *SE* = 0.014, *p* = 0.037) and that grit is not significantly associated with multitasking (*B* = −0.259, *SE* = 0.274, *p* = 0.344). More importantly, the cross-level interaction was significant (*B* = 0.048, *SE* = 0.022, *p* = 0.028), indicating that the relationship of the autonomy score and multitasking is different for people depending on their level of grit. As in Model 1 and 2, the variance components for autonomy and grit were significant (autonomy: *B* = 56.094, *SE* = 7.094, *p* < 0.001; grit: *B* = −0.379, *SE* = 0.062, *p* < 0.001).

Hypothesis H3 states that the cross level interaction effect is qualified by grit attenuating the effects of lower autonomy scores. In other words, we hypothesized that the multitasking behavior of grittier students is relatively independent from their current motivation, whereas students low in grit are particularly prone to multitask when their motivation is controlled. To visually assess if this is what the interaction effect represents, we divided all 930 situations into high vs. low AS and high vs. low grit using a median split. The median autonomy score was −1. Therefore, the low autonomy category consists of 467 study situations with autonomy scores between −1 and −18 (*M* = −5.46; *SD* = 3.61) and the high autonomy category consists of 463 study situations with autonomy scores between 0 and +18 (*M* = 6.36; *SD* = 5.43). As the median split divided autonomy scores between −1 and 0, the low autonomy category consists exclusively of situations with controlled motivation.

The median of grit across all situations was 3.50. Note that although grit is conceptualized as a trait measure and every participant reported grit only once, we used the median of the 930 situations and not of the 88 participants, holding the procedure parallel across both median splits. This procedure provides us with two categories of similar sample sizes concerning the situations, which can be used for visual illustration. The low grit category consists of 491 study situations with grit scores between 1.25 and 3.5 (*M* = 2.95; *SD* = 0.44), the high grit category consists of 439 study situations with grit scores between 3.63 and 4.75 (*M* = 3.89; *SD* = 0.24). The low grit group consisted of more students (*n* = 53), than the high grit group (*n* = 35), pointing in the direction that grittier students may have reported more study situations per person than their less gritty peers.

**Figure 1:**
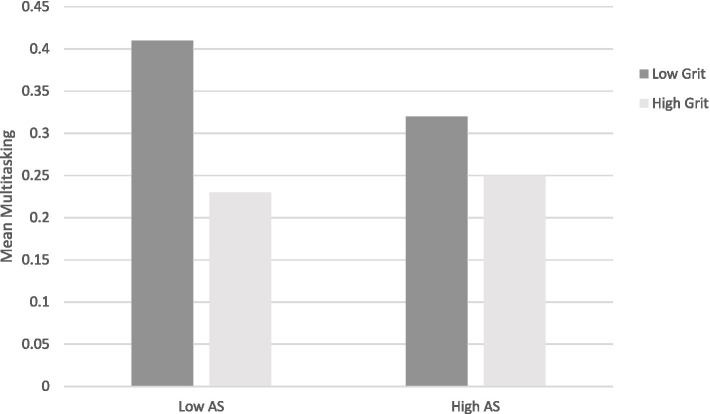
Illustrating multitasking behavior amongst grittier (light gray) and less gritty (dark gray) students in situations with controlled **(left)** or autonomous **(right)** motivation.

Figure 1 illustrates that, as predicted, the multitasking behavior during studying is relatively independent of the current motivation for gritty people (*M* = 0.25; *SD* = 0.43 for high AS and *M* = 0.23; *SD* = 0.42 for low AS), with gritty people multitasking 25% of their study time when motivation is autonomous and 23% of their study time when motivation is controlled. On the other hand, the multitasking likelihood of less gritty people fluctuates more with situational motivation (*M* = 0.32; *SD* = 0.47 for high AS and *M* = 0.41; *SD* = 0.5 for low AS), with less gritty students multitasking 32% of their time when the situational motivation is autonomous but 41% of the time when motivation is controlled.

## Discussion

### Theoretical implications

In the current study we investigated multitasking during studying using the experience sampling method. We found that students engage in additional activities during studying frequently, with approximately one third of all study activities being accompanied by an additional activity. Most additional activities come from the leisure domain and are social and/or media related (for similar results see Shih, 2013; Bachmann et al., 2018).

Firstly and consistent with our predictions, we found that students are particularly prone to multitask in situations when studying is motivated in a controlled way. According to self-determination theory autonomous motivation is predictive for many advantages in the academic sphere, such as higher engagement and more persistence (e.g., Grolnick and Ryan, 1987; Vansteenkiste et al., 2004; Froiland and Worrell, 2016; Manganelli et al., 2019). The current study widens these results by showing that autonomous motivation during studying is also predictive for less multitasking. These results are especially relevant because multitasking always implies branching off resources such as time and attention away from the main activity studying and into another activity, leading to declines in academic performance (Kraushaar and Novak, 2010; Clayson and Haley, 2013; Sana et al., 2013). Within the field of multitasking research, potential reasons behind multitasking have received more speculative (Judd and Kennedy, 2011; Adler and Benbunan-Fich, 2013) than empirical attention (Wang and Tchernev, 2012; Calderwood et al., 2014). The current study contributes to multitasking research by showing that one potential reason for multitasking lies in the situational motivation for the main activity.

Secondly, and contrary to our predictions, we did not find a significant main effect of grit predicting multitasking likelihood, i.e., gritty students did not multitask significantly less *per se* in comparison to less gritty students. Although the results did not yield significance it is noteworthy that the results point in the expected direction, and the interaction effect was significant. The null results from this main effect should be interpreted with some caution as one reason for them may lie in the power of the study. Nevertheless, the non-significance of the results indicates that the association of multitasking and grit is not as strong as we had hypothesized. One reason for this could be that grit is conceptualized to predict the successful accomplishment of long term, superordinate goals (Duckworth and Gross, 2014) and multitasking is a relatively short term phenomenon (Calderwood et al., 2014; Kim et al., 2019).

Lastly, we found a cross level interaction effect of grit and motivation. Consistent with our hypotheses, results indicate that the association of situational motivation with multitasking is strongest for students whose grit scores are low: While less gritty students multitask more when their current motivation is controlled, grittier students multitasking depends less on their current motivation. These results indicate that for regulating behavior in study situations, motivational factors and personal traits are both of relevance, in an interactive way.

In a perfect world in which study motivation is always autonomous, grit would not be linked to the proneness to multitask. In the real world however, study motivation is not always autonomous but sometimes rather controlled. In our sample in more than half of all sampled study activities motivation were controlled. And in these controlled study situations grit seems to have acted as a regulatory resource, helping gritty students to refrain from multitasking. This is congruent with reasoning of SDT researchers that motivation interacts with other regulatory resources (Moller et al., 2006).

When integrating these findings into the grit and SDT literature further, one has to address that earlier research often focused on the question, if motivational factors mediate the link of grit with outcome variables such as achievement, and well-being (Jin and Kim, 2017; Hong and Lee, 2019; Lozano-Jiménez et al., 2021); or research focused on the question if grit or motivational factors are more suitable for predicting achievement (Muenks et al., 2018; Steinmayr et al., 2018), thereby construeing motivation and grit as competing constructs to explain behavaiour and success. Although these research questions are interesting in their own right, we believe that a focus on the interaction between the two constructs is just as important in order to get a clearer understanding when and under which conditions different variables become important (for similar reasoning see Bonanno and Burton, 2013; Scholer and Miele, 2016). This could also contribute to cultivating a view within psychology that different constructs and variables are not so much competitors, but instead acknowledging that different constructs may be important in different situations and people. This view seems particularly relevant when trying to make use of theoretical findings in a real world context.

### Practical implications

A holistic view on academia acknowledges that in some situations study motivation is not autonomous; especially when studying for externally set goals such as writing an exam. In these situations students and teachers may find themselves unable to further internalize and optimize motivation, but instead students may need to persevere through these phases of controlled and suboptimal motivation. In these situations gritty students have one advantage – they focus their attention on their study activity, i.e., they are more likely to monotask, whereas less gritty students tend to multitask more, which likely leads to a drop in concentration and performance (e.g., Arnell and Duncan, 2002; Clayson and Haley, 2013). These findings support the recommendations for teachers at universities to support autonomous motivation (Ryan and Deci, 2017; Reeve and Cheon, 2021) and the development of grit (Duckworth, 2022).

However, there is also a potential disadvantage of grit which needs mentioning here. Research on SDT suggests prolonged exposure to high controlled and low autonomous motivation can negatively impact vitality, performance and well-being (Sheldon, 2011; Ryan and Deci, 2017). While multitasking can hinder learning (e.g., Bellur et al., 2015; May and Elder, 2018), multitasking can sometimes be a sign that a student is exploring other interests, which may indicate a mismatch between their studies and their personal inclinations (e.g., Wiradhany et al., 2021). For example, consider a student pressured into studying law. They multitask frequently, which could lead them to discover a more fitting interest and simultaneously highlight their misalignment with the law course. In this case, excessive grit may hinder multitasking and thereby hinder the realization that the current course was an unsuitable choice, potentially leading down an unfulfilling career path (Brandstätter and Bernecker, 2022).

Therefore, both motivation and grit are crucial for navigating the complexities of student learning. While grit helps persevere through controlled motivation phases, we cannot rule out that unchecked grit could hinder course correction (Brandstätter and Bernecker, 2022).

### Limitations and future research

The current study has several limitations. The most obvious limitation is that the experience sampling method does not allow for causal interferences. Future research could try to design an experiment in which study motivation is manipulated, for example by varying the autonomy support of teachers (Cheon et al., 2012), and collect data on multitasking. In this study we did not find a main effect for grit and multitasking. One potential reason may lie in the time frames of both constructs – multitasking being a very quick phenomenon and grit taking a long term perspective. However, because of the significant interaction effect, it is unreasonable to assume that grit and multitasking while studying are totally unrelated. Future research could investigate this connection further. Also, future research could investigate if high levels of grit really increase the risk of disengaging with unsuitable goals too late (Brandstätter and Bernecker, 2022).

Furthermore, our results encourage further research into the interaction effects between motivation and self-control. Imagine these two constructs not as independent forces, but as teammates in a complex game. Our results, along with the broader field of research, suggest that the “motivation players” are essential in propelling the game forward, maintaining momentum and stay on track (e.g., Ryan and Deci, 2017; Bachmann et al., 2024). However, when these players encounter obstacles or experience waning enthusiasm, the “grit players” become crucial. Their perseverance helps the team navigate challenges and maintain focus even during a dry spell. Research on the interactive effects of different constructs such as motivation and grit could help seeing these constructs not so much as competitors but as different players on a field, whereby every player has their chance to shine in different scenes of the game.

## Conclusion

The aim of the current study was to get a better understanding of why students multitask so frequently during studying. Only days before an upcoming exam, students participated in our ESM study, reporting momentary activity, multitasking and motivation. In a pre-test students also reported grit. Results indicate that situational motivation predicts multitasking likelihood, with more autonomous motivation being associated with less multitasking. These results give yet another reason to support students’ autonomous motivation wherever possible, as this seems to make it easier for students to refrain from multitasking and focus on their study activity instead. Also, we found a cross level interaction effect, showing that the situational motivation is particularly strongly related to multitasking for those students who score low on grit. In contrast, grittier students multitasking behavior stays more constant, not depending as much on their situational motivation. Supporting students improve their grit could help them make their multitasking behavior less dependent on situational variabilities such as autonomous motivation. However, as high grit may also increase the risk of engaging in unsuitable goals too long, the recommendation to increase grit if possible, is a more cautious one that the recommendation to support autonomous motivation.

## Data availability statement

The original contributions presented in the study are included in the article/supplementary material, further inquiries can be directed to the corresponding author.

## Ethics statement

The studies involving humans were approved by Ethik-Kommission der Universität Bielefeld. The studies were conducted in accordance with the local legislation and institutional requirements. The participants provided their written informed consent to participate in this study.

## Author contributions

OB: Conceptualization, Data curation, Formal analysis, Investigation, Methodology, Software, Visualization, Writing – original draft, Writing – review & editing. CG: Conceptualization, Data curation, Funding acquisition, Investigation, Methodology, Project administration, Resources, Supervision, Writing – review & editing. SF: Conceptualization, Funding acquisition, Project administration, Resources, Supervision, Writing – review & editing.
